# Optimizing aortic arch branch cannulation in acute type A dissection surgery: a minimally invasive approach

**DOI:** 10.3389/fcvm.2025.1549736

**Published:** 2025-05-16

**Authors:** Lin Xia, Ying Lyu, Xiong Xiao, Zhonglu Yang, Yuguang Ge, Bin Wang, Yu Liu, Hui Jiang

**Affiliations:** ^1^ Department of Cardiovascular Surgery, General Hospital of Northern Theater Command, Shenyang, Liaoning, China; ^2^ Department of Cardiopulmonary Bypass, Tianjin Chest Hospital, Tianjin, China

**Keywords:** acute type A aortic dissection, minimally invasive surgery, arterial cannulation, aortic arch branch perfusion, single upper hemisternotomy

## Abstract

**Background:**

The optimal cannulation strategy for acute type A aortic dissection (ATAAD) surgery via a minimally invasive approach remains a topic of debate. This study aimed to compare the feasibility and safety of different aortic arch branch cannulation techniques using a single upper hemisternotomy.

**Methods:**

A retrospective analysis was performed on 207 patients with ATAAD who underwent total arch replacement combined with frozen elephant trunk techniques between December 2019 and July 2023. Patients were categorized into four groups based on the cannulation site: IA group (innominate artery, *n* = 174), LCA group (left carotid artery, *n* = 21), RSA group (right subclavian artery, *n* = 5), and RCA group (right carotid artery, *n* = 7). Perioperative outcomes, including mortality, complications, and operative times, were compared using appropriate statistical methods.

**Results:**

A total of 207 patients were included and categorized into four groups based on the site of arterial cannulation: IA (*n* = 174), LCA (*n* = 21), RSA (*n* = 5), and RCA (*n* = 7). Baseline characteristics, including age and preoperative comorbidities, were comparable across the groups. Intraoperative metrics, such as cross-clamp time, circulatory arrest time, selective cerebral perfusion time, and cardiopulmonary bypass (CPB) time, showed no statistically significant differences. Although the CPB time was numerically shorter in the IA group, this difference was not significant (*p* > 0.05). Perioperative mortality occurred in 25 patients (12.1%), with no statistically significant differences among the groups (IA: 12.6%, LCA: 0%, RSA: 20.0%, RCA: 28.6%; *p* > 0.05). Postoperative clinical outcomes, including ventilator support duration, ICU stay, and hospital length of stay, were also similar across all groups.

**Conclusion:**

Aortic arch branch cannulation is a feasible and safe arterial perfusion strategy for ATAAD surgery via a minimally invasive single upper hemisternotomy. Among the options, the innominate artery demonstrated favorable outcomes and was not inferior to other arch vessels, and may be considered a suitable first choice when feasible.

## Introduction

Acute Type A aortic dissection (ATAAD) is a life-threatening cardiovascular emergency with an annual incidence of approximately 5.2 cases per million, characterized by high morbidity and mortality rates (1, 2). If untreated, the 48-h mortality rate for ATAAD exceeds 50%, underscoring the urgency of timely surgical intervention (3). Current standard surgical treatments typically involve total aortic arch replacement combined with frozen elephant trunk techniques, aimed at reducing complications and improving survival outcomes (4).

Arterial cannulation plays a pivotal role in ATAAD surgery, facilitating arterial perfusion and cerebral protection during circulatory arrest. Conventional strategies include femoral artery, axillary artery, and central cannulation, each with distinct advantages and limitations (5, 6). In recent years, innovative techniques such as apical and innominate artery cannulation have been explored to address the shortcomings of traditional methods (7).

The advent of minimally invasive surgery (MIS) has further transformed the management of ATAAD. Single upper hemisternotomy, utilizing J-shaped, L-shaped, V-shaped, or inverted T-shaped incisions, has emerged as a preferred approach for MIS due to reduced surgical trauma, shorter recovery times, and improved cosmetic outcomes (8, 9). However, common cannulation strategies in MIS, such as axillary and femoral artery cannulation, often require additional incisions, increasing the risk of infection and compromising aesthetic benefits. Wire-guided central cannulation, while safer in traditional settings, poses challenges for selective cerebral perfusion in minimally invasive contexts (10).

Cannulation via aortic arch branches offers a promising alternative. This approach eliminates the need for additional incisions, provides antegrade blood flow, and facilitates effective selective cerebral perfusion, making it particularly suited for MIS in ATAAD (3). Since 2017, our center has accumulated substantial experience with aortic arch branch cannulation in minimally invasive ATAAD surgeries, allowing for a comprehensive evaluation of its feasibility and safety under these conditions (11, 12).

This study aims to compare the outcomes of different aortic arch branch cannulation strategies in ATAAD surgery performed via a minimally invasive single upper hemisternotomy approach. By analyzing perioperative outcomes, we seek to determine the optimal cannulation strategy for this challenging clinical scenario.

## Methods

### Clinical data collection

A retrospective analysis was performed on patients who underwent minimally invasive surgery for acute Type A aortic dissection (ATAAD) at the General Hospital of the Northern Theater Command between December 2019 and July 2023. Perioperative clinical data were collected according to predefined inclusion and exclusion criteria, and patients were categorized based on the site of arterial cannulation.

Inclusion criteria included the confirmation of acute Stanford Type A aortic dissection involving the aortic arch using computed tomography angiography (CTA). Patients were excluded if they met any of the following conditions: (1) preoperative neurological complications such as cerebral hemorrhage or stroke; (2) preoperative malperfusion syndrome (4); or (3) concomitant diseases, including coronary heart disease, mitral valve disease, or congenital heart disease, requiring simultaneous surgical treatment through full sternotomy.

This study was approved by the hospital's ethics committee [Ethical Review K (2022)210], and informed consent was obtained from all patients.

### Surgical procedure

**Figure 1:**
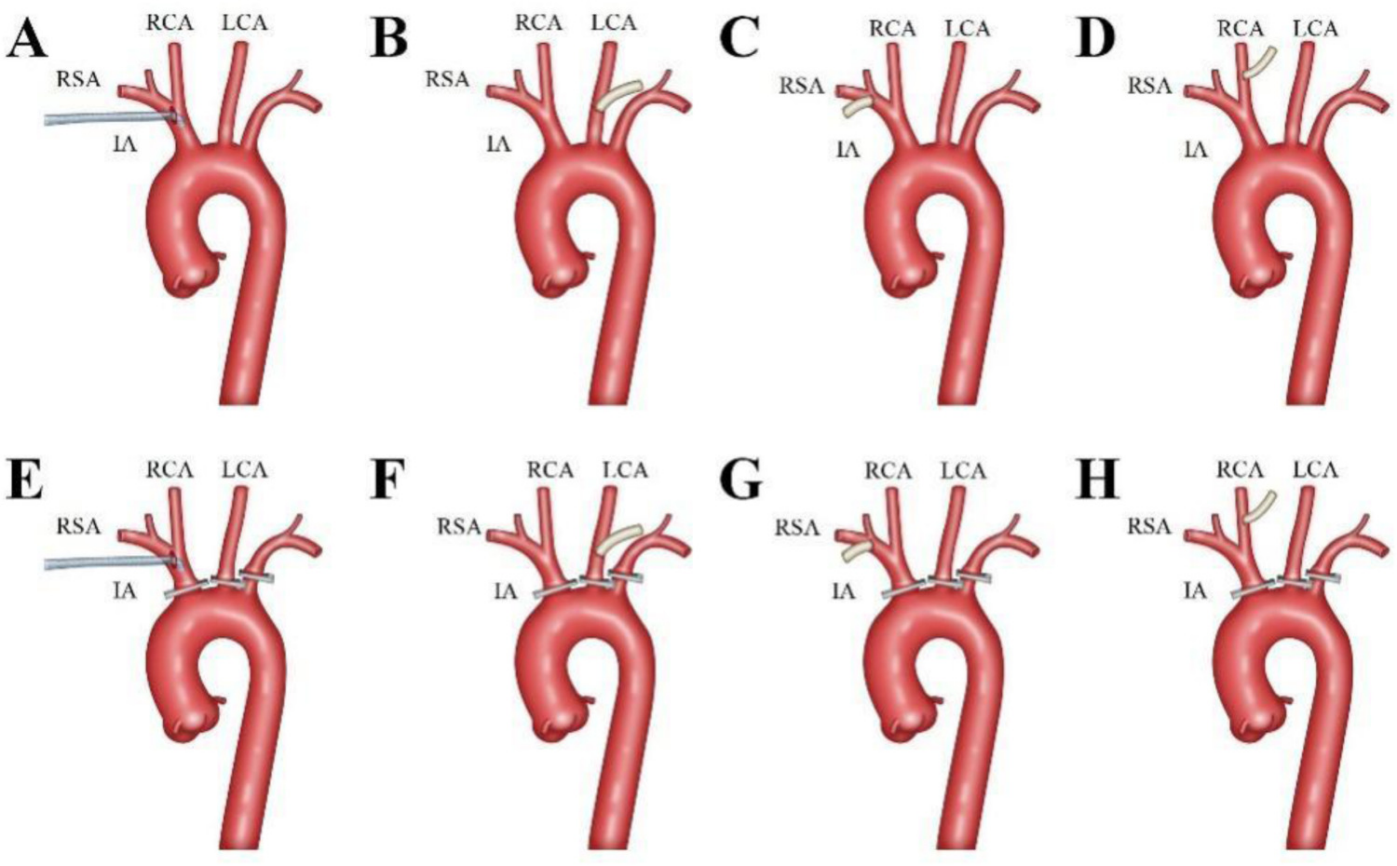
Aortic Arch Branches Cannulation Strategy. The innominate artery was the preferred cannulation site, with an 18Fr or 20Fr right angle arterial cannula (Longlaifu, Changzhou, China) inserted directly with its opening towards the heart **(A,E)**. The left carotid artery (LCA), right subclavian artery (RSA), and right carotid artery (RCA) were sequentially chosen as cannulation sites, with end-to-side anastomosis of a 0.8 cm diameter synthetic vessel to the LCA **(B,F)**, RSA **(C,G)**, and RCA **(D,H)**, respectively.

All patients included in this study underwent minimally invasive surgery via a single upper hemisternotomy approach for the repair of the proximal ascending aorta, total aortic arch replacement, and deployment of a frozen elephant trunk. The surgical technique has been described in detail in prior research and is summarized as follows:

An incision was made from the sternal notch to the fourth intercostal space. The sternum was sawed vertically either to the right (resulting in a J-shaped incision) or to the left (resulting in an L-shaped incision) (9). The aortic arch branches—including the innominate artery (IA), left carotid artery (LCA), and left subclavian artery (LSA)—were carefully dissected and examined. The site for arterial cannulation was determined based on whether these vessels were affected by the dissection (Figure 1).

The innominate artery was the preferred site for cannulation. A right-angled arterial cannula (18Fr or 20Fr, Longlaifu, Changzhou, China) was inserted directly, with its opening directed toward the heart. When the IA was involved in the dissection, alternative sites were selected in the following order: LCA, right subclavian artery (RSA), and right carotid artery (RCA). For these alternative sites, end-to-side anastomosis was performed using a synthetic vessel with a diameter of 0.8 cm (Maquet, La Ciotat Cedex, France), which was connected to the cardiopulmonary bypass (CPB) circuit via a connector.

Venous drainage was achieved through cannulation of the right atrium, and a left atrial drainage tube was placed through the right upper pulmonary vein. After CPB initiation, gradual cooling was employed. Following aortic clamping and cardiac arrest, the proximal ascending aorta was repaired. Selective bilateral antegrade cerebral perfusion was provided using a pump-controlled system (13).

When the target nasopharyngeal temperature was reached, either lower body circulatory arrest (28°C) (11) or brief circulatory arrest followed by lower body perfusion (31°C) (12) was utilized for descending aorta procedures. The surgery concluded with routine total aortic arch replacement, involving sequential anastomoses of the LCA, proximal ascending aorta, LSA, and IA.

### Statistical analysis

All statistical analyses were performed using SPSS version 22.0 (IBM Corp., Armonk, NY, USA). Continuous variables with a normal distribution are presented as mean ± standard deviation (SD) and were compared using one-way analysis of variance (ANOVA). Non-normally distributed continuous variables are expressed as medians with interquartile ranges (IQR, P25-P75) and were analyzed using the Kruskal–Wallis test. Categorical variables are presented as counts and percentages, and group comparisons were conducted using Fisher's exact test. A two-tailed *p*-value of <0.05 was considered statistically significant.

## Results

### Patient inclusion and grouping

**Figure 2:**
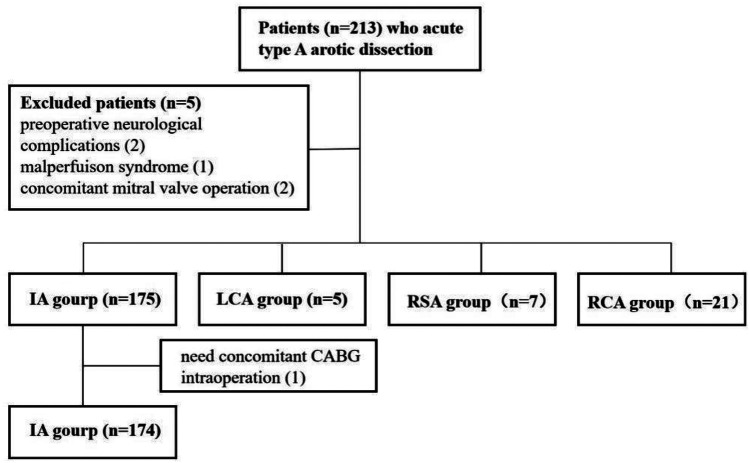
Patients enrolled based on inclusion and exclusion criteria.

The study initially included 213 patients. Six cases were excluded: two due to preoperative neurological complications, one due to preoperative malperfusion syndrome, two due to the need for concurrent mitral valve surgery, and one due to intraoperative discovery of coronary involvement requiring coronary artery bypass grafting. Consequently, 207 patients were included in the final analysis (Figure 2).

Patients were categorized based on the site of arterial cannulation into four groups: IA (*n* = 174), LCA (*n* = 21), RSA (*n* = 5), and RCA (*n* = 7). Baseline characteristics were comparable across all groups, with no statistically significant differences (Table 1).

**Table 1:** Preoperative factors of patients.

Indicators	IA group (*n* = 174)	LCA group (*n* = 5)	RSA group (*n* = 7)	RCA group (*n* = 21)	*P-*value
Age (years)	51.0 (43.0, 59.0)	52.0 (46.5, 58.5)	54.0 (45.0, 62.0)	55.0 (45.5, 64.5)	0.732
Male [*n* (%)]	128 (73.6)	3 (60.0)	5 (71.4)	15 (71.4)	0.851
Weight (kg)	79.0 (68.0, 90.0)	62.0 (58.0, 72.5)	85.0 (65.0, 95.0)	72.5 (65.0, 89.0)	0.070
Hypertension [*n* (%)]	5 (2.9)	0 (0.0)	0 (0.0)	1 (4.8)	0.652
Diabetes [*n* (%)]	83 (48.0)	0 (0.0)	4 (57.1)	10 (47.6)	0.193
Smoking History [*n* (%)]	7 (4.0)	0 (0.0)	0 (0.0)	2 (9.5)	0.567
LVEF (%)	59.0 （57.0, 60.0)	59.0 (56.5, 60.5)	58.0 (57.0, 60.0)	58.0 (57.0, 60.0)	0.913
Preoperative ALT (U/L)	20.9 (14.6, 33.7)	12.8 (11.6, 57.5)	35.3 (17.4, 43.8)	23.8 (15.3, 27.5)	0.434
Preoperative (AST) (U/L)	20.2 (15.1, 27.6)	25.4 (17.2, 49.6)	25.4 (22.3, 44.1)	19.4 (17.4, 32.3)	0.103
Preoperative UN (mmol/L)	6.0 (4.7, 7.6)	8.2 (4.9, 8.9)	6.0 (3.9, 6.3)	5.8 (4.3, 7.3)	0.415
Preoperative Cr (mmol/L)	68.9 (57.3, 89.2)	62.1 (44.8, 85.1)	56.7 (48.8, 73.7)	66.3 (47.0, 77.9)	0.267

LVEF, left ventricular ejection fraction; ALT, alanine aminotransferase; AST, aspartate aminotransferase; UN, urea nitrogen; Cr, creatinine.

### Intraoperative data

Intraoperative data are summarized in Table 2. There were no significant differences among the four groups in terms of CPB time, aortic clamping time, circulatory arrest time, selective cerebral perfusion time, or nadir temperatures (both nasopharyngeal and rectal).

**Table 2:** Intraoperative factors of patients.

Indicators	IA group (*n* = 174)	LCA group (*n* = 5)	RSA group (*n* = 7)	RCA group (*n* = 21)	*P*-value
Aortic valve repair [*n* (%)]	82 (47.1)	4 (80.0)	5 (71.4)	12 (57.1)	0.274
Aortic valve replacement [*n* (%)]	6 (3.4)	0 (0.0)	0 (0.0)	0 (0.0)	1.000
Bentall procedure [*n* (%)]	20 (11.5)	1 (20.0)	0 (0.0)	4 (19.0)	0.417
Wheat procedure [*n* (%)]	1 (0.6)	0 (0.0)	0 (0.0)	0 (0.0)	1.000
David procedure [*n* (%)]	4 (2.3)	0 (0.0)	0 (0.0)	0 (0.0)	1.000
Ascending Aorta replacement [*n* (%)]	136 (78.2)	4 (80.0)	6 (85.7)	16 (76.2)	0.969
Total arch + ETS [*n* (%)]	174 (100.0)	5 (100.0)	7 (100.0)	20 (95.2)	0.159
Bilateral cerebral perfusion [*n* (%)]	163 (93.7)	5 (100.0)	6 (85.7)	20 (95.2)	0.619
CPB time (min)	155.0 (137.0,180.0)	170.0 (156.0,188.5)	163.0 (134.0,179.0)	171.0 (158.5,196.5)	0.072
CA time (min)	6.0 (5.0,12.0)	11.0 (4.5,15.0)	6.0 (4.0,7.0)	7.0 (5.0,13.0)	0.330
ACC time (min)	88.5 (74.0,100.3)	97.0 (91.5,117.0)	96.0 (87.0,109.0)	86.0 (59.0,105.5)	0.280
SCP time (min)	30.0 (25.0, 36.3)	28.0 (23.5, 49.0)	32.0 (31.0, 39.0)	30.0 (25.0, 44.5)	0.560
MNT (℃)	29.5 (28.0, 30.4)	29.4 (28.1, 30.2)	31.6 (29.5, 32.3)	28.8 (27.6, 30.5)	0.069
MRT (℃)	31.0 (30.0, 32.0)	30.9 (30.5, 31.9)	31.0 (30.0, 32.0)	31.1 (30.9, 31.9)	0.589

ETS, elephant trunk stent; CPB, cardiopulmonary bypass; ACC, aortic cross-clamp; CA, circulatory arrest; SCP, selective cerebral perfusion; MNT, minimum nasopharyngeal temperature; MRT, minimum rectal temperature.

### Postoperative clinical outcomes

A total of 25 patients (12.1%) died during the perioperative period. This included 22 cases (12.6%) in the IA group, no deaths in the LCA group, 1 case (20.0%) in the RSA group, and 2 cases (28.6%) in the RCA group. While the RSA and RCA groups had numerically higher mortality rates, Fisher's exact test indicated no statistically significant differences among the groups.

Postoperative clinical metrics, including ventilator support duration, ICU stay, and hospital length of stay, were analyzed. No statistically significant differences were observed among the four groups (Table 3).

**Table 3:** Postoperative factors of patients.

Indicators	IA group (*n* = 174)	LCA group (*n* = 5)	RSA group (*n* = 7)	RCA group (*n* = 21)	*P*-value
Ventilation time (h)	38.08 (18.75, 88.56)	21.08 (16.83, 1,180.42)	64.33 (41.00, 83.00)	22.33 (17.29, 88.25)	0.611
ICU stay time (h)	46.46 (39.31, 112.02)	85.17 (28.54, 1,926.42)	89.25 (48.75, 161.17)	45.58 (19.04, 113.04)	0.301
Reoperation for bleeding [*n* (%)]	1 (0.6)	0 (0.0)	0 (0.0)	0 (0.0)	1.000
Reventilation [*n* (%)]	7 (4.0)	0 (0.0)	2 (28.6)	0 (0.0)	0.073
Postoperative 24 h Drainage (ml)	200.00 (150.00, 270.00)	200.00 (185.00, 495.00)	240.00 (110.00, 350.00)	180.00 (150.00, 260.00)	0.660
Hospitalization time (d)	15.00 (11.00, 19.00)	11.00 (7.00, 13.00)	16.00 (10.00, 28.00)	17.00 (11.00, 19.50)	0.171
In-hospital deat [*n* (%)]	22 (12.6)	1 (20.0)	2 (28.6)	0 (0.0)	0.080
CRRT [*n* (%)]	14 (8.0)	0 (0.0)	1 (14.3)	2 (9.5)	0.720
Preoperative ALT (U/L)	25.670 (16.460, 41.478)	29.590 (14.585, 79.700)	49.230 (20.920, 170.910)	31.190 (20.300, 59.475)	0.285
Preoperative (AST) (U/L)	36.310 (26.833, 59.825)	34.830 (25.570, 99.010)	38.490 (26.320, 151.810)	46.910 (25.630, 64.560)	0.836
Preoperative UN (mmol/L)	13.125 (10.353, 16.065)	11.740 (8.990, 21.945)	14.860 (12.630, 19.790)	11.750 (9.160, 14.870)	0.174
Preoperative Cr (mmol/L)	119.455 (82.398, 167.225)	75.100 (62.410, 237.025)	172.930 (89.100, 274.180)	99.270 (64.445, 156.325)	0.262
Stroke [*n* (%)]	13 (7.5)	1 (20.0)	1 (14.3)	1 (4.8)	0.339
Postoperative transfusion [*n* (%)]	111 (63.8)	3 (60.0)	3 (42.9)	12 (57.1)	0.630

ICU, intensive care unit; CRRT, continuous renal replacement therapy; ALT, alanine aminotransferase; AST, aspartate aminotransferase; UN, urea nitrogen; Cr, creatinine.

## Discussion

This study suggests that the innominate artery is a suitable and effective site for cannulation in minimally invasive incisions for ATAAD, with the left carotid artery, right carotid artery, and right subclavian artery serving as viable alternatives. While the favorable outcomes observed with innominate artery cannulation support its use, it should be noted that patients in whom this approach was feasible may represent a lower-risk subset, which could introduce selection bias. The use of aortic arch branches as the primary strategy for arterial perfusion eliminates the need for additional incisions, thereby improving cosmetic outcomes and reducing the risk of infection, while maintaining procedural feasibility and safety. Conventional ATAAD surgeries are widely regarded as highly invasive procedures associated with significant bleeding risks (14, 15), making patient survival the foremost priority. Although right axillary and femoral artery cannulation remain common due to their rapid setup, these methods require additional incisions, which can increase complications and compromise cosmetic outcomes. With advancements in minimally invasive techniques, the feasibility of performing ATAAD surgeries through single upper hemisternotomy incisions has been increasingly explored by cardiovascular surgery centers (16, 17). This approach aligns with clinical goals to minimize invasiveness while improving cosmetic outcomes.

This retrospective study of 207 ATAAD cases demonstrated the feasibility of using aortic arch branches as cannulation sites for arterial perfusion. Specifically, 174 cases utilized innominate artery (IA) cannulation, 21 used left carotid artery (LCA) cannulation, 5 adopted right subclavian artery (RSA) cannulation, and 7 employed right carotid artery (RCA) cannulation. These findings highlight the suitability of aortic arch branches for arterial perfusion in the majority of ATAAD surgeries, with IA cannulation being the preferred choice, followed by LCA, RSA, and RCA.

The IA group showed shorter cardiopulmonary bypass (CPB) and aortic clamping times compared to the other groups, although the differences were not statistically significant. This can be attributed to the direct cannulation technique used in the IA group, whereas the other groups required synthetic vessel anastomosis. Additionally, the severity of dissection involvement in the IA observed in other groups may have contributed to the lack of statistical significance. While these results suggest that cannulation site selection has limited impact on overall surgical outcomes, the study supports the feasibility and practicality of using aortic arch branches as arterial perfusion sites in minimally invasive ATAAD surgeries.

The selection of arterial perfusion cannulation is guided by several critical considerations, as outlined in reference (11). These include: (1) assessment of aortic arch branch involvement using preoperative computed tomography angiography (CTA); (2) intraoperative evaluation of the degree of branch involvement; (3) determination of branch diameter; (4) suitability for selective cerebral perfusion; and (5) ease of exposure and cannulation of the target vessel. Based on these criteria, the innominate artery is identified as the optimal site for arterial perfusion.

The innominate artery offers several distinct advantages: (1) it has the largest diameter among the aortic arch branches, ensuring adequate flow; (2) it provides straightforward exposure and facilitates direct cannulation; and (3) it supports the direct execution of selective cerebral perfusion. From our experience, a right-angled arterial cannula, oriented toward the heart, is preferred for direct insertion. Orienting the cannula toward the brain can result in excessive perfusion, known as luxury perfusion, which may have adverse effects. Given the relatively narrower diameter of the innominate artery compared to the ascending aorta, an 18Fr or 20Fr right-angled cannula is typically chosen based on intraoperative findings. Special attention is given to avoiding the use of an oversized cannula, which could occlude the innominate artery and compromise cerebral blood supply.

In cases where the innominate artery is affected or unsuitable for cannulation, alternative sites, including the left common carotid artery (LCA), right subclavian artery (RSA), and right carotid artery (RCA), are considered. Due to their smaller diameters, direct cannulation of these vessels is often impractical. Instead, synthetic vessels are utilized for end-to-side anastomosis, enabling connection to the cardiopulmonary bypass (CPB) system through a connector. This approach effectively achieves the desired arterial perfusion while addressing the limitations of smaller vessel diameters.

The most significant advantage of utilizing aortic arch branches as conduits for arterial perfusion lies in eliminating the need for additional surgical incisions (18). Compared to femoral artery cannulation, this approach offers several benefits: (1) mitigation of retrograde dissection expansion caused by retrograde perfusion; (2) prevention of blood supply to the false lumen, thereby avoiding retrograde blood flow that could dislodge atherosclerotic plaques and reduce the risk of stroke; (3) avoidance of lower limb ischemia resulting from cannulation; and (4) facilitation of antegrade selective cerebral perfusion, which is essential for optimal surgical outcomes (19, 20).

Similarly, when compared to axillary artery cannulation, aortic arch branch cannulation provides additional advantages (6, 21): (1) avoidance of the complex axillary artery dissection procedure, thereby reducing the risk of complications such as brachial plexus nerve injury; and (2) prevention of catastrophic complications associated with vascular anomalies, such as misdiagnosis of an aberrant right subclavian artery.

However, aortic arch branch cannulation is not without limitations. The restricted diameter of the target vessels may limit cannulation options and result in constrained flow rates. Nonetheless, during the cooling phase of cardiopulmonary bypass (CPB), the demand for high flow rates decreases, and this limitation becomes less critical. During the rewarming phase, a four-branch synthetic vessel is used for perfusion, ensuring adequate flow to meet metabolic demands.

Our clinical experience confirms that during total arch replacement performed through a minimally invasive single upper hemisternotomy, the simultaneous application of pump-controlled selective cerebral perfusion (13) and antegrade lower body perfusion (22) is both safe and feasible. Despite occupying a portion of the surgical field, these procedures can be effectively conducted through the minimally invasive incision, demonstrating the practicality of this approach in achieving successful surgical outcomes.

### Limitations

This study has several inherent limitations: (1) As a retrospective investigation, the study is subject to selection bias, potentially influencing the results. (2) Being a single-center study with a relatively small sample size, the findings may be susceptible to confounding factors and may not be generalizable to broader populations. (3) The limited number of patients in the non-IA groups reduces the statistical power of between-group comparisons. This imbalance reflects institutional practice and is difficult to avoid in a retrospective single-center study, but it realistically represents real-world clinical decision-making. (4) In addition, patients who underwent innominate artery cannulation may have represented a lower-risk cohort with more favorable anatomy, particularly with respect to cerebral malperfusion. This potential selection bias could have influenced the observed clinical outcomes and should be considered when interpreting the results. Future multicenter studies with larger sample sizes are warranted to validate these findings and provide more robust conclusions.

## Conclusion

This study demonstrates that aortic arch branch cannulation is a feasible and effective arterial perfusion strategy for acute Type A aortic dissection surgeries performed via a minimally invasive single upper hemisternotomy. This technique eliminates the need for additional incisions, enhances cosmetic outcomes, and maintains procedural safety. Among the aortic arch branches, the innominate artery was associated with favorable surgical outcomes and ease of access, and may be considered a suitable first-line option when feasible, though potential selection bias should be acknowledged.

## Data Availability

The raw data supporting the conclusions of this article will be made available by the authors, without undue reservation.
